# Helpful self-management strategies to cope with enduring depression from the patients’ point of view: a concept map study

**DOI:** 10.1186/s12888-014-0331-7

**Published:** 2014-12-13

**Authors:** Rosa A van Grieken, Anneloes CE Kirkenier, Maarten WJ Koeter, Aart H Schene

**Affiliations:** Department of Psychiatry, Program for Mood Disorders, Academic Medical Center, University of Amsterdam, Meibergdreef 5, 1105 AZ Amsterdam, The Netherlands; Department of Psychiatry, Radboud University Medical Center, Reinier Postlaan 6, 6500 HB Nijmegen, The Netherlands; Donders Institute for Brain, Cognition and Behavior, Radboud University, Geert Grooteplein Noord 21, 6525 EZ Nijmegen, the Netherlands

**Keywords:** Self-management, Depression, Patients’ Perspective

## Abstract

**Background:**

Despite the development of various self-management programmes that attempt to ameliorate symptoms of patients with chronic major depressive disorder (MDD), little is known about what these patients perceive as helpful in their struggle during daily live. The present study aims to explore what patients believe they can do themselves to cope with enduring MDD besides professional treatment, and which self-management strategies patients perceive as being most helpful to cope with their MDD.

**Methods:**

We used concept mapping, a method specifically designed for the conceptualisation of a specific subject, in this case patients’ point of view (n = 25) on helpful self-management strategies in their coping with enduring MDD. A purposive sample of participants was invited at the Academic Medical Center and through requests on several MDD-patient websites in the Netherlands. Participants generated strategies in focus group discussions which were successively clustered on a two-dimensional concept map by hierarchical cluster analysis.

**Results:**

Fifty strategies were perceived as helpful. They were combined into three meta-clusters each comprising two clusters: *A focus on the depression* (sub clusters: Being aware that my depression needs active coping and Active coping with professional treatment); *An active lifestyle* (sub clusters: Active self-care, structure and planning and Free time activities) and *Participation in everyday social life* (sub clusters: Social engagement and Work-related activities).

**Conclusions:**

MDD patients believe they can use various strategies to cope with enduring MDD in daily life. Although current developments in e-health occur, patients emphasise on face-to-face treatments and long-term relations, being engaged in social and working life, and involving their family, friends, colleagues and clinicians in their disease management. Our findings may help clinicians to improve their knowledge about what patients consider beneficial to cope with enduring MDD and to incorporate these suggested self-management strategies in their treatments.

## Background

Major depressive disorder (MDD) is a major cause of disability worldwide [1]. Poor recovery occurs in 10-20% of the MDD patients [2] and can have various reasons, e.g. not seeking treatment, a preference to manage MDD on their own, insufficient adherence to or no effective use of treatment and non- or partial response to sequential treatments resulting in treatment resistant depression (TRD) [3-5]. To cope with residual symptoms of MDD, various recent developed psychological therapies (e.g. acceptance and commitment therapy (ACT) and mindfulness-based cognitive therapy (MBCT)) [6] have shifted the focus of these therapies from achieving complete recovery to more realistic goals such as acceptance and coping with the changed health circumstances [7].

To date, clinicians and politicians are shifting from paternalistic to partnership models of care for chronic diseases. The latter states that the patient should be an active partner in the treatment, involving his or her knowledge of the disease throughout the treatment process [8]. Various chronic disease management models and (internet-based) self-management programmes have been developed [9-11], aimed at improving the confidence of patients with chronic diseases, empowerment, health-related quality of life, and changing the behaviour to ameliorate symptoms and stop disease progression. Such programmes are also a widespread strategy to reduce health care costs in expanding populations with chronic diseases [12].

The self-management literature is expanding rapidly and although chronic MDD patients should, according to stepped care approaches described in guidelines [13], be treated with “high intensity” interventions (e.g. cognitive behavioral therapy or antidepressant medication), literature suggests that chronic MDD patients also derive comparable clinical benefit from “low-intensity” interventions (e.g. self-management) [14]. However, some authors raise questions about the effectiveness of self-management programmes because findings vary from promising to benefits that are transient and do not sustain [15]. Most research in this field has mainly focused on sub threshold depression and primary care patients [16] and most self-management programmes and therapies have been developed and led by professionals. To our knowledge, no study has examined how chronic MDD patients themselves perceive self-management and what they exactly consider helpful in their struggle with enduring MDD in daily live, whether they receive treatment or not. This knowledge may be a valuable addition to the literature and to further development of self-management treatments [17].

In our previous study [18], we found a wide range of self-management strategies that patients perceive as helpful. That study, however, focused on *recovery from* MDD, not on *coping with* enduring MDD. As part two of our self-management project we conducted an explorative study among MDD patients with enduring depressive symptoms in spite of optimal antidepressant treatments. With this study, we aimed to explore what patients believe they can do themselves in daily life to cope with enduring MDD besides professional treatment.

Patients’ views on what they actually do to cope with depression may be distorted due to depressive symptomatology, related negative cognitions and maladaptive appraisals. However, by focusing on what they believe they can do themselves to cope with depression, we think we can assess their ideas about what possibly works for them. So we consider depressed patients to be able to assess their own capacities, also realizing that they often have high demands towards how they should behave. Their perspectives about what works for them may help clinicians to open the conversation about what is really useful to cope with depression.

We aimed to answer the following questions: 1) What do patients believe they can do themselves to cope with enduring depression besides professional treatment? 2) Which main self-management strategy themes can be derived from their point of view? 3) Which of these strategies do these patients perceive as most helpful? This knowledge may contribute to advice and clinical decisions about what works for whom in treating MDD.

## Methods

### Participants

Participants were eligible for the study if they met the following criteria: 1) Diagnosed with a major depressive episode (MDE) according to Diagnostic and Statistical Manual of Mental Disorders (DSM-IV-TR) criteria, 2) A score of seven or higher on the Hamilton Depression Rating Scale (HDRS) [19], indicating a current MDE, and 3) Received at least two different treatments (two different classes of antidepressants and/or psychotherapy) from a psychiatrist or a psychologist for the current MDE with a poor or unsatisfactory response. ACEK assessed the participants. Exclusion criteria for the study were: age younger than 18 years, insufficient command of the Dutch language, bipolar disorder, MDD with psychotic characteristics, a terminal disease, mental retardation or suicidality. Because many (chronic) depressive patients suffer from suicidal thoughts (score 1, 2 or 3 on the third item of the HDRS), we decided that only those scoring 4 (suicide attempts) would be excluded.

We purposively sampled participants from different parts of the Netherlands who had a variety of treatment experiences, ranging from outpatient- to inpatient treatment and from medication contacts to various types of (group) psychotherapy by posting a request for study participation on several MDD patient-websites. In addition, we recruited participants by invitations at the Program for Mood Disorders at the Academic Medical Center (AMC). In total twenty-five patients participated in the concept map. Table 1 displays characteristics of these twenty-five participants. Sixteen participants took part in the strategies generation stage and the prioritizing and sorting stage. They were successively allocated to two focus groups of 5 participants and one focus group of 6 participants. This number of participants and focus groups was sufficient to reach saturation in the exploration of strategies. An additional nine participants only took part in the prioritizing and sorting stage, with the aim to give more power to the quantitative results. The Medical Ethics Research Committee of the Academic Medical Center considered ethical approval not necessary and approved the oral informed consent we obtained.

**Table 1:** **Characteristics of the concept map participants (n = 25)**

**Characteristic**	
*Gender (n)*	
Male	10
Female	15
*Age (years)*	
Mean (SD)	49 (11.1)
Range	28-67
*Ethnicity (n)*	
Dutch	25
Other	0
*Employment (n)*	
> 20 hours/week	6
< 20 hours/week	7
None	12
*HDRS (n)*	
Mild (score 7-17)	9
Moderate (score 18-24)	14
Severe (score ≥25)	2
Treatment history setting (n)*	
Psychiatric hospital admission	5
Day care treatment	12
Outpatient treatment	15
Type of treatment (n)*	
Antidepressants	21
Psychotherapy	17
Electroconvulsive therapy	3

*Including overlap.

### Concept mapping

We used concept mapping to address the research questions. We have chosen this method for two reasons: First, concept mapping is specifically designed for the conceptualisation of a specific subject [20,21], in this case patients’ experiences and perceptions of their coping with enduring MDD. Second, because concept mapping uses group processes to encourage participants to bring up more ideas between each other than would emerge in individual interviews.

Concept mapping consists of five stages:

1. Preparation
  Participants were encouraged to brainstorm in 1,5 hour focus group sessions about their experiences and perceptions concerning the central question: ‘*What, from your experience, is the best you can do yourself to cope with your depression, if the depression is enduring besides treatment?’* The sessions were guided by a trained researcher (ACEK).
2. Strategies generation
  Coping strategies were generated around this central question and visualized for the group via a digital projector. The researcher (re)formulated the strategies with the help of the participants untill they were clear to everyone. Next, two researchers (ACEK and MWJK) combined the strategies from the focus groups and removed overlapping strategies, resulting in a final strategy list.
3. Prioritizing and sorting strategies
  Each individual participant had to prioritize the strategies of the final list by dividing them into 5 groups of equal size. Group 1 was defined as ‘least important’ for the coping with enduring MDD and group 5 as ‘most important’. In addition, each participant was asked to sort the strategies based on their own idea of whether they belonged to the same category in 5 to 12 groups, each group containing 2 to 20 strategies. These numbers were chosen to guarantee enough heterogeneity between the statement groups. The list of strategies was presented to most participants in a Word-document and structuring was done with a computer. Participants who only took part in the ‘prioritizing and sorting stage’ received the strategies printed onto cards and sent as a homework assignment.
4. Statistical analysis
  Prioritizing and sorting data from all participants were analysed with the computer programme ‘Ariadne’, specifically designed to analyse these kind of data [22]. First, the strategies were positioned in a two-dimensional map with a X and Y axis based on multidimensional scaling. In this map, the distance between two strategies represents how often the participants sorted them together in one group. Second, the individual strategies were clustered using hierarchical cluster analysis [20].
5. Interpretation of the concept map
  This cluster analysis offers solutions with 2 to 18 clusters. Two researchers (RAvG and MWJK) reviewed each of these 17-computer-generated cluster solutions and the best interpretable cluster solution was selected after consensus with ACEK and AHS, based on the interpretability. Subsequently, the relative importance of the individual strategies and the clusters was calculated based on the mean participants’ prioritizing score. In addition, for each strategy, the percentage of participants that considered it important or very important (i.e. a priority score of 4 or 5) was calculated. Each cluster received a descriptive name based on the content and importance of the strategies comprising this cluster [20].

## Results

### Self-management strategies and main themes

The participants produced a total of 98 strategies. The third focus group hardly introduced any new strategy and so we concluded that we had reached data saturation. Removal of overlapping strategies resulted in a final list of 50 strategies for the prioritizing and sorting stage.

**Figure 1:**
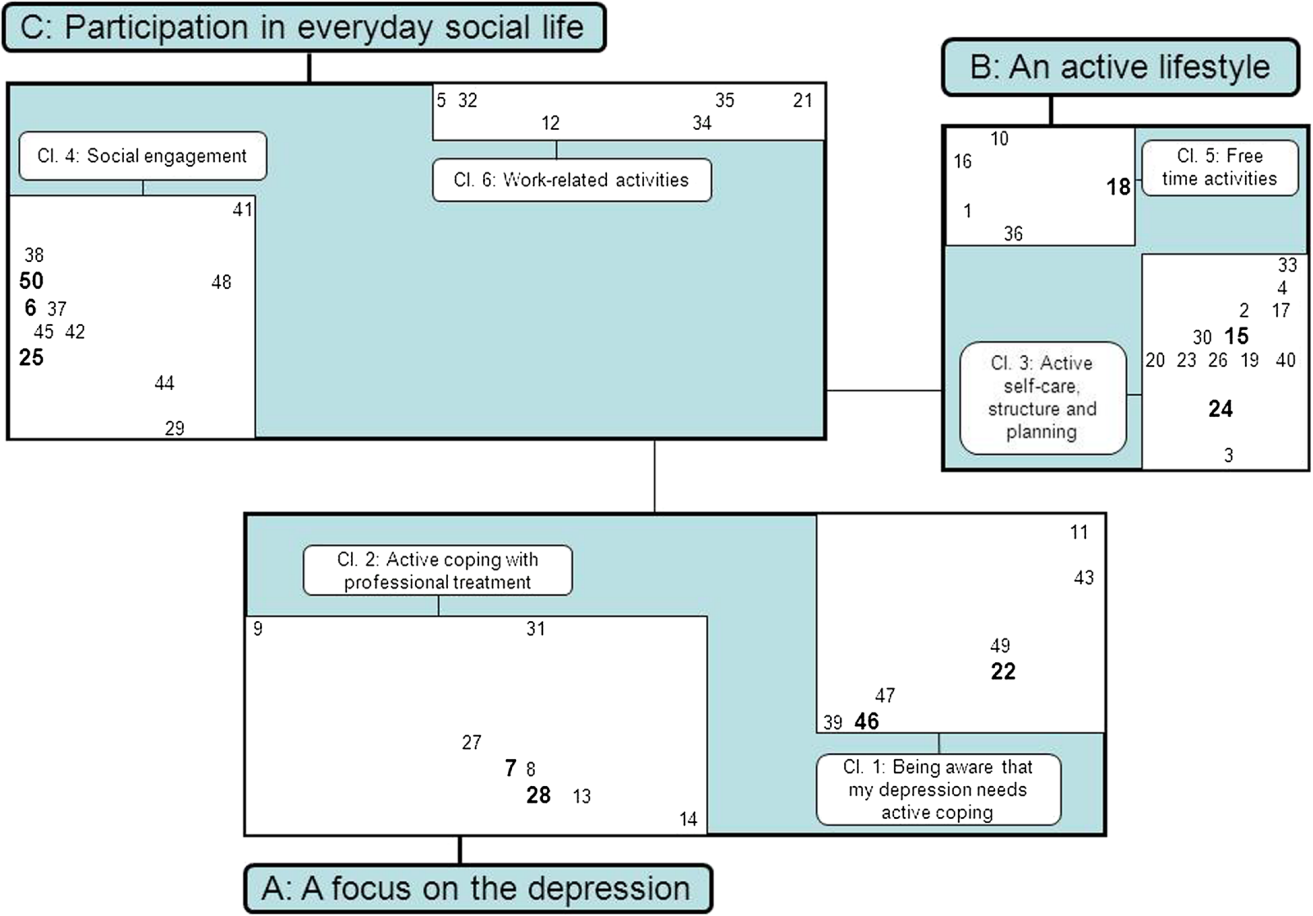
**Concept map: Helpful self-management strategies in coping with enduring MDD from the patients’ point of view; Strategies, clusters and meta-clusters.** The map displays the 50 strategies presented as numbers, the six clusters (1-6) and three meta-clusters **(A-C)**.

Figure 1 shows the 50 strategies positioned as dots in a concept map and combined into three meta-clusters and six clusters. The proximity of these strategies (dots) is based on which strategies were more likely to have been sorted in the same group by the participant. The six-cluster solution was considered the best interpretable one according to the content of the strategies belonging to these clusters. A solution with less clusters comprised clusters that were too heterogeneous and a solution with more clusters meant that either 1 item clusters emerged or that good interpretable clusters were divided into two or more smaller clusters. Additional meaning about their interrelation appeared when we combined these six clusters into three meta-clusters.

Moreover, the circular position of the strategies with its empty centre shows that there is little ambiguity about the positioning among participants: they agree about the reciprocal relation of the strategies. We numbered the clusters in order of their perceived importance (e.g. the mean priority score of all strategies within this cluster). Cluster 1, ‘A focus on the depression’, is considered by participants as the most helpful cluster in coping with MDD. Names given to the clusters were based on the strategies within the cluster with most emphasis placed on strategies considered more important by the participants. An overview of the clusters with their corresponding strategies, sorted by patients’ priority score, is presented in Table 2. The concept map resulted in the following three meta-clusters and six clusters:

**Table 2:** **Strategies per (meta-)cluster with priority**

**Number**	**Cluster theme with strategies**	**Mean (SD)** ^**1**^	**Priority ≥ 4** ^**2**^ **(%)**
**Meta-cluster A: A focus on the depression**			
**Cluster 1**	**Being aware that my depression needs active coping**	**3.34** ^**3**^	
46	Take the signals of my depression seriously	4.08 (1.19)	68
22	Acknowledging that depression is a disease	3.96 (1.48)	72
11	Take more and more decisions myself again	3.44 (1.21)	44
39	Engaging in a structured form of meditation (e.g. yoga, mindfulness)	3.16 (2.37)	44
43	Do my best not to avoid issues	3.16 (2.89)	36
49	Taking my medication at a fixed place and/or time	2.84 (2.53)	36
47	Using a positive mantra	2.76 (2.18)	32
**Cluster 2**	**Active coping with professional treatment**	**3.33**	
28	Maintaining long-term professional support	4.00 (1.20)	76
7	Finding a therapist with whom I feel connection	3.68 (1.26)	56
13	Finding a type of treatment that suits me	3.40 (1.28)	56
31	Taking warnings from others about increasing of my depression seriously, even if I do not notice it myself	3.36 (1.59)	48
9	Making sure I have telephone support when needed from family/friends/an organisation	3.32 (1.34)	48
14	Finding information about my depression	3.00 (1.44)	40
27	Making sure there is professional support when using my medication	3.00 (2.00)	40
8	Asking my therapist for explanation about medication	2.96 (1.80)	44
**Meta-cluster B: An active lifestyle**			
**Cluster 3**	**Active self-care, structure and planning**	**3.00**	
24	Ensuring enough rest to avoid exhaustion through over-exertion	3.68 (1.58)	60
15	Setting realistic short-term goals	3.56 (1.29)	56
26	Making sure to have a good day/night rhythm	3.40 (2.16)	64
3	Ignoring the tiredness associated with my depression	3.12 (1.55)	40
19	Get dressed every day	3.12 (2.75)	52
17	Engaging consciously and unstressed in activities	3.04 (1.96)	44
4	Making an adjusted activity schedule	3.00 (1.60)	28
40	Keeping a diary	2.80 (2.80)	40
2	Take a shower every morning	2.72 (2.28)	36
30	Healthy eating	2.72 (2.28)	36
23	Making sure that I will be awakened every morning	2.68 (2.78)	40
20	Making plans for the future	2.68 (1.66)	28
33	Taking every opportunity to tide the house	2.48 (1.29)	20
**Cluster 5**	**Free time activities**	**2.74** ^**3**^	
18	Leaving the house regularly	3.72 (1.56)	60
10	Engaging in sports activities	3.20 (1.36)	48
16	Exploring creative hobbies	2.84 (1.97)	32
1	Develop an unused talent	2.28 (1.64)	16
36	Writing an own weblog	1.68 (1.34)	12
**Meta-cluster C: Participation in everyday social life**			
**Cluster 4**	**Social engagement**	**2.94**	
6	Informing close family/friends about my depression	3.64 (1.03)	52
50	Explaining my depression to family/friends	3.52 (1.45)	48
25	Involving close family/friends in my treatment	3.48 (1.45)	60
37	Meet with the friends with whom I can be myself	3.40 (1.52)	52
45	Discussing depression with those I trust in order to have support nearby	3.40 (1.52)	60
42	Ask my family/friends to actively keep in contact with me	3.36 (1.99)	48
38	Discussing my depression with trusted people to fetch predjudices	2.80 (1.68)	32
44	Seeking contact with fellow sufferers	2.64 (1.83)	24
29	Searching out my family background	2.28 (2.12)	24
48	Planning a holiday adapted to my possibilities in consultation with my family/friends	2.16 (1.49)	16
41	Searching for contact with new people in my life without obligations	1.64 (1.03)	4
**Cluster 6**	**Work-related activities**	**2.47**	
32	Explaining my depression to my manager	2.64 (1.75)	24
5	Explaining my depression to close colleagues	2.60 (1.44)	24
34	Finding out which activities are achievable	2.52 (1.61)	28
12	Provide backup from colleagues for work that is too much for me	2.40 (1.52)	20
35	Slowly start (volunteering) work	2.36 (1.51)	16
21	Finding occupations (e.g. volunteering)	2.32 (1.34)	16

^1^Mean priority per strategy with standard deviation (SD).

^2^Percentage of patients that prioritize the strategy as important/very important (4 or 5).

^3^Cluster mean score.

Bold values indicate the 10 most important strategies.

Meta-cluster A: ‘*A focus on the depression*’ is situated at the lower region of our concept map and contains 15 strategies grouped into the following two clusters:

1. *Being aware that my depression needs active coping:* Comprising strategies focussing on active coping to be considered as helpful, such as take the signals of depression seriously (st. 46) and engaging in a structured form of meditation (st. 39).
2. *Active coping with professional treatment:* strategies in this cluster indicate that maintaining long-term professional support (st. 28), with a therapist with whom the participant feels connected (st. 7), as well as keeping informed about depression (st. 14) and medication (st. 8) is perceived as important.

Meta-cluster B: ‘*An active lifestyle’* is situated at the upper right region of the concept map and is composed of 18 strategies grouped into the following two clusters:

3. *Active self-care, structure and planning:* These strategies indicate the importance to maintain daily self-care, such as get dressed every day (st. 19), healthy eating (st. 30) and structure and planning, such as making sure to have a good day/night rhythm (st. 26) and making an adjusted activity schedule (st. 4).
5. *Free time activities:* Strategies in this cluster relate to engaging in depression-independent free time activities, such as sports activities (st.10) and exploring creative hobbies (st. 16).

Meta-cluster C: *‘Participation in everyday social life’* is situated at the upper left region of the concept map and contains 17 strategies grouped into the following two clusters:

4. *Social engagement:* Participants perceive social contacts to be very helpful in coping with their depression and consider strategies such as informing and explaining the depression to close family and friends (st. 6 and 50) and their involvement in treatment (st. 25) as being important.
6. *Work-related activities:* This cluster emphasizes the importance of investing in work-related activities, ranging from finding occupations for participants without a job (st. 21), to explaining the depression to my manager (st. 32) and close colleagues (st. 5) while having a job.

### Most helpful strategies

Table 3 displays the 10 strategies perceived as most helpful in coping with enduring MDD. Two of these strategies are from the cluster ‘*Being aware that my depression needs active coping’*, two from the cluster ‘*Active coping with professional treatment’*, two from the cluster ‘*Active self-care, structure and planning’*, three from the cluster ‘*Social engagement*’ and one from the cluster ‘*Free time activities*’.

**Table 3:** **The 10 strategies perceived most helpful in coping with enduring MDD**

**Cluster**	**Number**	**Mean (SD)** ^**1**^	**Strategies**
1	46	4.08 (1.19)	Take the signals of my depression seriously
2	28	4.00 (1.20)	Maintaining long-term professional support
1	22	3.96 (1.48)	Acknowledging that depression is a disease
5	18	3.72 (1.56)	Leaving the house regularly
2	7	3.68 (1.26)	Finding a therapist with whom I feel connection
3	24	3.68 (1.58)	Ensuring enough rest to avoid exhaustion through over-exertion
4	6	3.64 (1.03)	Informing close family/friends about my depression
3	15	3.56 (1.29)	Setting realistic short-term goals
4	50	3.52 (1.45)	Explaining my depression to family/friends
4	25	3.48 (1.45)	Involving close family/friends in my treatment

^1^Mean priority per strategy with standard deviation (SD).

## Discussion

The present study shows that patients believe they can use a variety of helpful strategies to cope with a mostly moderate to severe type of MDD that has not or only partly responded to at least two different treatments. These coping strategies can be summarized into three main themes: *A focus on the depression,* comprising ‘Being aware that my depression needs active coping’ and ‘Active coping with professional treatment’; *An active lifestyle,* comprising ‘Active self-care, structure and planning’ and ‘Free time activities’ and; *Participation in everyday social life,* including ‘Social engagement’ and ‘Work-related activities’. Although this wide range of themes indicate that coping with MDD involves various aspects of daily life, patients consider the first theme*, A focus on the depression*, with its two clusters emphasizing an active coping with depression and professional treatment, both with nearly the same mean priority scores, to be the *most* helpful. The most important strategies from the patients’ point of view are also from these two clusters: ‘Take the signals of my depression seriously’ and ‘Maintaining long-term professional support’.

Furthermore, among the 50 strategies only two strategies, ‘Writing an own weblog’ (st. 36) and ‘Searching for contact with new people in my life without obligations’ (st. 41), are perceived as *least* important for coping with enduring MDD, with a mean priority score below 2. All other strategies have a higher mean priority score. These results suggest that, although we did not examine whether the perceived helpful strategies actually helped in practice, patients believe and perceive that they can do a lot themselves during their daily life in coping with their enduring MDD.

Striking about these findings is that the strategies perceived to be helpful are all the opposite of avoidance behaviour, a clinical pattern so often seen in MDD [23]. Instead of withdrawing from daily life, staying in bed all day or not answering the phone, the patients in our study emphasize the usefulness of staying engaged in social and working life, by involving their family, friends and colleagues in their disease. This could be the result of effective (previous) treatment experiences and underlines the importance of behavioural activation in MDD treatment from the patients’ perspective.

Another important result is that, although the patients in this study need to cope with their enduring MDD and with the disappointment that previous treatments did not result in full recovery, they still consider the focus on the disease and on a professional treatment to be helpful. Maybe patients do not want to give up their hope to achieve recovery. However, new developments in treatment strategies for patients with long term or chronic MDD focus on acceptance more than recovery (e.g. ACT and MBCT) [6]. Our results indicate that clinicians should explore how patients experience MDD treatment, and they should involve the patients’ perspective and concerns about treatment in decision-making because new developments in treatment focusing on acceptance may not fit to what patients perceive as helpful: a focus on recovery.

Interestingly, patients did not mention internet strategies, such as e-health or online peer support, to be helpful for their coping whilst over the past 10 years there has been increasing interest in internet-based (self-help) interventions [24,25]. One could hypothesize that selection bias might have caused this result; that the participants in our study did not have experience with those types of treatment, or that mainly participants were selected that had treatment experience with a clinician, and therefore did not mention these kind of strategies. On the other hand, these results may also indicate that patients prefer face-to-face contacts with professionals, family, friends and colleagues above the internet.

Compared to the results of our previous study [18], in which we identified helpful self-management strategies from the patients’ perspective in the recovery from (non-chronic) MDD, there seem to be little differences between strategies perceived as helpful for *recovery* and strategies perceived as helpful for *coping* with enduring MDD. With respect to recovery strategies, activity-related strategies seem to be considered slightly more important, while for coping, the emphasis is more on social engagement. In addition, substantial overlap exists between the strategies we found and the strategies perceived to be helpful for sub threshold depression [16]. This suggests that the strategies we have found may be helpful for all MDD patients, irrespective of their severity stage (sub-threshold, mild or severe MDD), and therefore they can be used in primary as well as secondary care treatments.

### Implications

Our study gives a comprehensive overview of self-management strategies perceived as helpful in coping with enduring MDD by patients. This may help clinicians to improve their knowledge and understanding about what patients consider beneficial to cope with enduring MDD in their daily life. They may discuss these strategies with their patients during treatments and emphasize *those* strategies perceived as *most helpful* by other patients. This may stimulate MDD patients to change their behaviour in such a way that they can improve their coping with the disease. However, this also requires a new role for clinicians in line with a partnership model of treatment. Our results may further contribute to the evidence, effectiveness and current developments of self-management programmes for MDD with an added value: focus on the patients’ perspective. According to the current innovative shift towards e-health programmes with the patient managing its treatment, it is important to emphasis on a blended formula including the combination of e-health with face-to-face treatments and long-term relations.

### Strengths and limitations

This study has several strengths: first, the present study is, to our knowledge, the first to explore the patients’ point of view on helpful self-management strategies in their coping with enduring MDD. Second, the credibility of the results is supported by the selection of a broad range of participants from different parts of the Netherlands who had a variety of treatment experiences. Next, the concept mapping method is a favourable combination of brainstorm sessions in *groups* and sorting assignments eliciting *individual* reflection, in which participants can prioritize and asses the experiences of other participants.

However, our study also has some limitations. First, we included a purposive sample of twenty-five participants, although this is in line with the protocol for concept mapping [26], the external generalizability of our findings may be modest because we might have missed specific subgroups within depression. Nevertheless, our sample was appropriate for our purpose; an exploration of helpful strategies. Second, participants were selected if they had MDD and poor or unsatisfactory response to at least two different treatments; we considered it not necessary to specifically select participants with chronic depression or TRD for our purpose: to explore perceptions of patients with *enduring* MDD. Finally, this study focused on strategies considered important for coping by patients. Whether what patients perceive as helpful, really works in practice is a question for future quantitative research. Depressed patients may have a distorted view because of their negative cognitions caused by their depressed mood. However, in our experience, most patients have high demands about how they have to behave and are able to give their opinion about how to cope with their depression without the negative cognitions about their own capacities. Moreover, even if patients have some distorted view about what strategies work for them or not, these results are still relevant, because if patients believe in strategies that are absolutely wrong according to clinicians’ view or evidence, this must be used by clinicians to find connection with their patients and must become a target of conversation between patient and clinician.

## Conclusion

In conclusion, the present study shows that MDD patients believe they can use various strategies to cope with enduring MDD in daily life. Although current developments in e-health occur, patients emphasise on face-to-face treatments and long-term relations, being engaged in social and working life, and involving their family, friends, colleagues and clinicians in their disease management. This adds to the understanding of depression management and may help clinicians to incorporate these suggested self-management strategies from patients’ perspectives in their treatments.
